# EasyGraph: A multifunctional, cross-platform, and effective library for interdisciplinary network analysis

**DOI:** 10.1016/j.patter.2023.100839

**Published:** 2023-09-05

**Authors:** Min Gao, Zheng Li, Ruichen Li, Chenhao Cui, Xinyuan Chen, Bodian Ye, Yupeng Li, Weiwei Gu, Qingyuan Gong, Xin Wang, Yang Chen

**Affiliations:** 1 Shanghai Key Lab of Intelligent Information Processing, School of Computer Science, Fudan University, Shanghai, China; 2 Department of Interactive Media, Hong Kong Baptist University, Hong Kong, China; 3 College of Information Science and Technology, Beijing University of Chemical Technology, Beijing, China

**Keywords:** interdisciplinary network analysis, multiprocessing optimization, hybrid Python/C++ programming, structural hole theory

## Abstract

Networks are powerful tools for representing the relationships and interactions between entities in various disciplines. However, existing network analysis tools and packages either lack powerful functionality or are not scalable for large networks. In this descriptor, we present EasyGraph, an open-source network analysis library that supports several network data formats and powerful network mining algorithms. EasyGraph provides excellent operating efficiency through a hybrid Python/C++ implementation and multiprocessing optimization. It is applicable to various disciplines and can handle large-scale networks. We demonstrate the effectiveness and efficiency of EasyGraph by applying crucial metrics and algorithms to random and real-world networks in domains such as physics, chemistry, and biology. The results demonstrate that EasyGraph improves the network analysis efficiency for users and reduces the difficulty of conducting large-scale network analysis. Overall, it is a comprehensive and efficient open-source tool for interdisciplinary network analysis.

## Highlights

• EasyGraph is a Python library for network analysis across various disciplines
• EasyGraph adopts the advantage of multiprocessing to enhance processing efficiency
• EasyGraph provides an easy-to-use Python interface
• EasyGraph achieves an efficient implementation with C++ modules

## The bigger picture

Networks are effective for representing relationships between entities across a range of disciplines, and network analysis techniques are widely used for understanding various types of complex networks, e.g., social networks, biological networks, transportation networks. Network analysis tasks, such as community detection, centrality analysis, and network visualization, play important roles in many disciplines. Existing network analysis tools, however, lack efficiency in analyzing massive network data or may not provide comprehensive analysis functions, which limits their practical applicability. We present EasyGraph, an open-source library that supports many network data formats and covers important functions like structural hole spanner detection and network embedding. Notably, we have optimized several key functions for enhanced efficiency. We believe that EasyGraph is a powerful tool for dealing with major analytical tasks in complex networks across various domains.

## Graphical abstract

**Figure:**
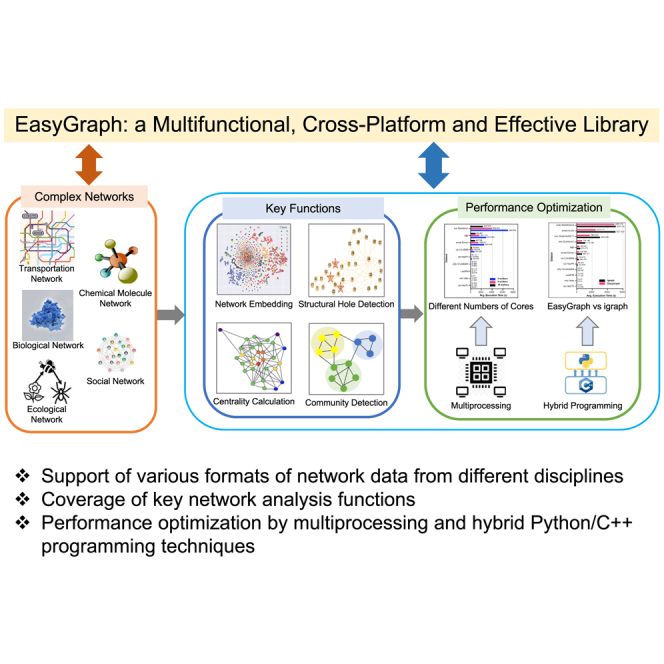

## Teaser

Network analysis is a cornerstone of network science research. Here, the authors present EasyGraph, an open-source network analysis library that supports a series of important interdisciplinary network analysis functions. It enables researchers and practitioners to explore various properties and applications of different types of networks. EasyGraph uses two main optimization techniques to speed up several critical functions. Comparative studies show that EasyGraph outperforms igraph and NetworkX, two other representative network analysis libraries.

## Introduction

A network, which is also known as a graph, is a powerful data structure for modeling complex relationships and interactions between entities. The term “graph” refers to the graph of graph theory in mathematics. We will use both “network” and “graph” throughout this descriptor. Vertices or nodes within a graph are linked together in pairs, facilitating the representation of various scenarios across disciplines such as sociology, physics, biology, mathematics, psychology, finance, and computer science.^1^^,^^2^^,^^3^^,^^4^^,^^5^^,^^6^ The concept of a network provides a unifying framework for exploring and understanding important scientific questions in multiple disciplines. Network analysis, which is also called network science,^7^ is a critical research methodology for investigating the statistical properties,^8^^,^^9^ topological structures,^10^^,^^11^ and evolution models^12^^,^^13^ of networks that span a wide range of disciplines. To accelerate research in various disciplines using network analysis, it is imperative to develop a flexible, functional, and efficient package that meets the needs of users.

With the increasing size of networks, analyzing massive network data effectively becomes a major challenge. Additionally, addressing the diverse needs of various disciplines necessitates providing a comprehensive range of functionalities for network analysis. To address these challenges, many tools and open-source libraries have been developed, including NetworkX (https://networkx.org/),^14^^,^^15^^,^^16^ SNAP (http://snap.stanford.edu/),^17^^,^^18^^,^^19^^,^^20^ igraph (https://igraph.org/),^21^^,^^22^^,^^23^^,^^24^ graph-tool (https://graph-tool.skewed.de/), Gephi (https://gephi.org/),^25^^,^^26^^,^^27^ Cytoscape (https://cytoscape.org/),^28^^,^^29^ and GraphVis (https://networkrepository.com/graphvis.php).^30^^,^^31^^,^^32^^,^^33^ These tools offer a range of features and capabilities to support network analysis for research across a variety of disciplines. Although these tools and packages cover most functions of network analysis, including centrality measurement,^34^^,^^35^^,^^36^ influence maximization,^37^^,^^38^ community detection,^3^^,^^39^ and basic network characteristics,^40^^,^^41^^,^^42^ they are limited in terms of several important functions. For example, NetworkX and igraph currently do not support network embedding. Likewise, graph-tool and SNAP do not support structural hole identification. However, these functions have proven to be critical for addressing significant theoretical problems such as the investigation of gene regulatory function^43^ and for many applications such as anomaly detection^44^ and friend recommendation,^45^ as well as molecular property prediction.^46^^,^^47^ Therefore, users have to implement the corresponding functions for network analysis from scratch. These limitations may result in diminished research efficiency and difficulty in reproducing and benchmarking the research results. Furthermore, existing tools and packages support only a limited number of network formats (e.g., NetworkX supports five types of network formats, and SNAP only supports two types of network formats) and are inefficient at performing network analysis on large-scale networks.^48^ Particularly, it is time consuming for some existing tools (such as NetworkX) to execute algorithms with high computational complexity (such as betweenness centrality calculation) on large-scale networks. Therefore, it is very difficult for users to carry out experiments and verify the feasibility and validity of their research methods in practical network settings.

**Figure 1:**
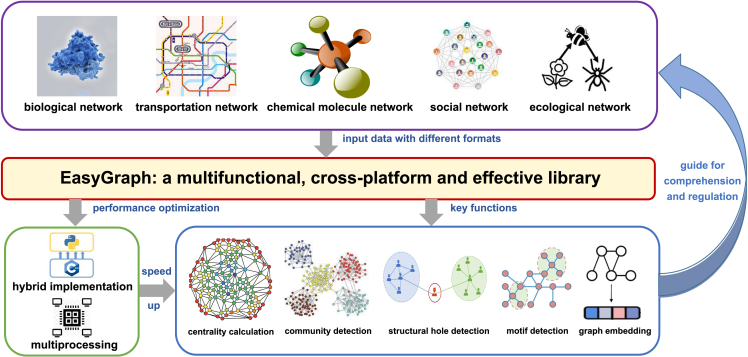
Framework of EasyGraph

To fill these gaps, we designed and implemented EasyGraph as an ease-of-use toolkit for interdisciplinary network analysis, computation, and representation of crucial graph properties covering multiple graph data formats from different disciplines. EasyGraph is available at https://github.com/easy-graph/Easy-Graph. We encourage readers to fork this repository, submit pull requests, and open new issues through the GitHub interface. We have made EasyGraph an open-source project, and we will make further improvements based on user needs and feedback during the development process. EasyGraph is implemented in Python for ease of access and provides the option for multiprocessing and computation on a C++ backend. EasyGraph aims to accelerate the exploration of significant graph properties and important applications of graphs from various disciplines. An overview of the EasyGraph framework is presented in Figure 1. EasyGraph provides comprehensive functionality support for network analysis, computation, and representation. Therefore, it can assist users in different disciplines to comprehend complex phenomena and moderate network functions.

First, based on its strong compatibility, EasyGraph can flexibly model network data from different domains and support network data in a variety of formats. For example, the adjacency list format contains several lines with node labels, where the first node label is the source node and the other node label(s) is (are) the target node(s). Existing network analysis libraries only support a limited set of network formats (e.g., GraphML, GML), and there is no single library that could cover all mainstream graph formats across different domains. To fill this gap, we developed EasyGraph to support more diverse network data formats and achieve enhanced compatibility. We describe the available formats for network data below.

• GraphML format (http://graphml.graphdrawing.org/): this format describes a graph using XML tags and includes a GraphML element with three subelements: graph, node, and edge. The GraphML element defines a namespace by adding various XML attributes. The GraphML format is flexible for specific network data such as hypergraphs and hierarchical graphs. Therefore, this format can be widely found in many datasets.
• GML format (https://gephi.org/users/supported-graph-formats/gml-format/): the full name of this format is Graph Modeling Language. GML stores network data in a text format with simple syntax. This format is frequently used based on its flexibility for storing network data.
• Pickle format: this format contains a serialized byte stream of a hashable Python object. In this format, Python objects are preserved as nodes and edges. This format is supported by EasyGraph, NetworkX, and igraph.
• Pajek format: this is another text format that simplifies the representation of a graph’s structure in a text document. Each node has a distinct label, and edges are represented as pairs of nodes. Pajek also supports weighted graphs. This format is supported by EasyGraph, NetworkX, and igraph.
• GraphViz format (https://graphs.grevian.org/example): this format uses the DOT language to represent graphs. GraphViz uses standardized syntax to define graphs, where nodes, edges, and their attributes are covered if needed. This format is supported by EasyGraph, igraph, SNAP, and graph-tool.
• UCINET DL format (https://gephi.org/users/supported-graph-formats/ucinet-dl-format/): this format includes two subformats, full matrix and edge list. The former is suitable for small and dense graphs, and the latter is convenient for large and sparse graphs. Among the aforementioned network analysis libraries, this format is only supported by EasyGraph.
• GEXF format: the full name of this format is Graph Exchange XML Format. It is a representation format oriented toward the structure and dynamic evolution of data in complex networks. GEXF is found in many processes of graph data reading, writing, and transformation. It is now a mature and extensive graph format in various real-world scenarios. Among the aforementioned network analysis libraries, this format is only supported by EasyGraph.

Second, EasyGraph encapsulates a massive set of metrics and algorithms for network analysis and representation for the study of different disciplines. Among these, representative functions include centrality measurement, community detection, structural hole spanner (SH spanner) detection,^49^^,^^50^^,^^51^^,^^52^^,^^53^ motif detection,^54^^,^^55^^,^^56^ and network embedding.^57^^,^^58^^,^^59^^,^^60^^,^^61^ Because EasyGraph integrates these key functions, users from various domains can perform the operations they need without having to switch between tools. Subsequently, this will not only minimize switching costs but also enhance research efficiency. We highlight one special function, namely SH spanner detection, below.

In addition to being an important theoretical contribution, the SH theory offers significant application guidance for various disciplines.^52^^,^^62^ The SH theory was first proposed in 1992 by Burt.^62^ According to Burt, bridging positions play a critical role in information dissemination through social networks. The individuals occupying such positions, called SH spanners, act as brokers among communities, thereby facilitating the flow of information and communication among different groups. SH spanner analysis, an important concept frequently used in network science^52^^,^^63^^,^^64^^,^^65^ and innovation studies,^66^^,^^67^ refers to the identification of individuals or entities within a network that serve as bridges between separate groups or communities within the network. SH spanners occupy unique positions that span the gaps between otherwise disconnected groups, so the analysis of SH spanners and their connected communities can provide insights into how information, resources, and influence flow through a network. Several recent works in different disciplines have presented novel explorations based on SH spanner analysis. Li et al.^63^ studied the role of SHs in information diffusion. Saglietto et al.^64^ reviewed the literature on SHs utilizing a bibliometric methodology. According to Yang et al.,^65^ the shift to firm-wide remote work made it more difficult for workers who serve as SH spanners to benefit from new network connections. Lin et al.^52^ presented the development of applications for the analysis of SH spanners among enterprise settings, information diffusion, software development, mobile applications, and machine learning tasks. Zaheer et al.^67^ found that innovative firms can achieve a performance boost if they bridge SHs. Ahuja^66^ described a theoretical framework for studying the influence of SHs on innovation. As indicated above, the study of SH spanner analysis has been widely applied in different disciplines because SH spanners have a special location advantage in a network.

**Figure 2:**
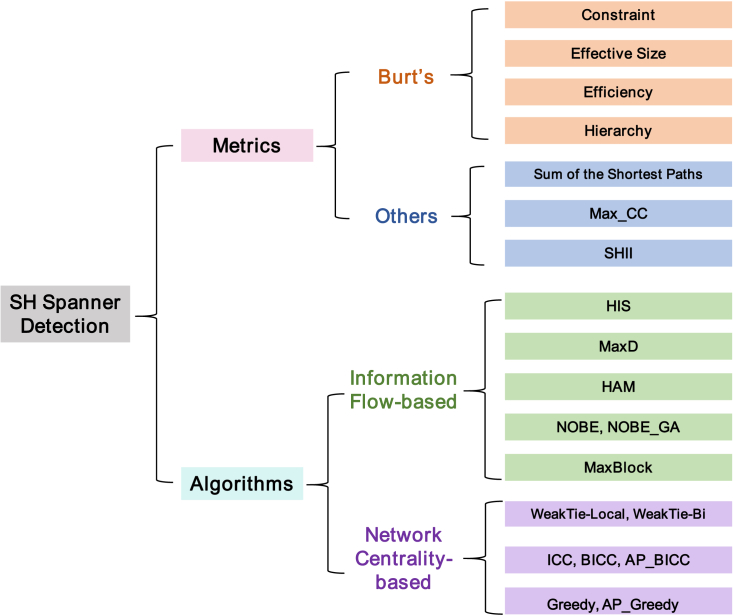
SH spanner detection methods and metrics implemented in EasyGraph

In recent years, a growing number of methods and algorithms have been proposed to detect SH spanners. However, these approaches are often implemented in different programming languages or platforms by their authors, and some are not open source. As a result, users face difficulties in accessing and using these methods conveniently. To address this issue, EasyGraph provides a collection of implemented SH spanner detection methods, including HIS, MaxD, HAM, NOBE, and NOBE_GA, as well as MaxBlock for information flow-based algorithms and WeakTie-Local, WeakTie-Bi, ICC, BICC, AP_BICC, Greedy, and AP_Greedy for network centrality-based algorithms, based on previous studies.^52^ By consolidating these methods in EasyGraph, we aim to provide an effective library for users to understand the role of SH spanners in network analysis. Our library includes the most commonly used SH spanner detection methods, enabling users to choose methods that are well suited to their particular research requirements. Overall, EasyGraph fills a gap in the availability of SH spanner detection methods, which provides users with a unified and reliable solution for their network analysis requirements. Figure 2 presents the implemented metrics and algorithms for SH spanner detection in EasyGraph.

Finally, EasyGraph utilizes multiprocessing techniques and hybrid programming to optimize the performance of the algorithms it provides. Specifically, EasyGraph leverages multiprocessing techniques to accelerate the computation of some vital metrics such as closeness centrality, betweenness centrality, and Burt’s SH metrics, which could be calculated in a distributed manner. Furthermore, EasyGraph utilizes hybrid implementations (i.e., two versions of base network classes are implemented in Python and C++) to reduce the running time of various methods for network analysis.

Because it is a dynamically typed language with a lack of concurrency, Python is relatively slow compared with other mainstream programming languages, such as C++. Accordingly, most pure Python packages (e.g., NetworkX) may yield poor computational performance, especially when handling large-scale data. To alleviate this issue, we implemented multiprocessing optimizations for several commonly used representative metrics. The computation of these metrics could be further optimized through multiprocessing because they can be computed individually and without interdependencies. For example, all of Burt’s metrics for SH spanners can be optimized through multiprocessing techniques because each metric is a property or value of each node that can be calculated individually.

Although the technology for multiprocessing optimization improves the running speed of various network analysis methods, many others still suffer from Python’s inherent low efficiency, particularly for large-scale networks. To address this issue, we adopted a hybrid implementation strategy that leverages the efficiency of C++ and the ease of use of Python. Specifically, EasyGraph utilizes pybind11, which is a lightweight header-only library that seamlessly integrates C++11 and Python, as a framework for hybrid programming. EasyGraph has pure C++ implementations of several important methods (e.g., betweenness centrality and closeness centrality), and it still offers a simple Python interface for users to call these methods. Additionally, the base network classes and their basic operations are implemented in C++. The usage of specific algorithms can be achieved by calling Python API interfaces. Our intuition is that all network analysis algorithms are composed of numerous basic network class operations (e.g., degree centrality calculation and finding neighbors). This is why this type of hybrid approach can make a significant difference.

Overall, EasyGraph offers significant advantages in terms of efficiency, important functions, and ease of use, making it a valuable tool for network analysis across different disciplines.

## Results

In this section, we present the comparisons between EasyGraph and other existing network analysis tools to demonstrate our tool’s superiority in terms of both functional comprehensiveness and performance enhancement.

### Multiple types of network input/output (I/O) and extensive functions

We investigated the formats of network data supported by existing network analysis tools, and the results are presented in Table 1. We selected representative network analysis tools, including NetworkX, igraph, SNAP, graph-tool, Gephi, and Cytoscape. In Table 1, one can see that there are rich formats available for representing network data. This may be attributed to the fact that there is no uniform specification in the processes of data collection and storage. Additionally, one can see EasyGraph’s superiority in terms of handling different formats of network data, which is user friendly for users who possess network data in different formats.

**Table 1:** Comparison of network analysis tools in terms of supporting different network I/O types

Network I/O types	Tools						
	EasyGraph	NetworkX	igraph	SNAP	graph-tool	Gephi	Cytoscape
Edge List	✓	✓	✓	✓	–	✓	–
GraphML	✓	✓	✓	–	✓	✓	✓
GML	✓	✓	✓	–	✓	✓	✓
Pickle	✓	✓	✓	–	✓	–	–
Pajek	✓	✓	✓	–	–	✓	–
GraphViz	✓	–	✓	✓	✓	✓	–
UCINET DL	✓	–	–	–	–	✓	–
GEXF	✓	–	–	–	–	✓	–

Because EasyGraph implements both the SH spanner detection methods and SH-related metrics, we can use these metrics for fair comparisons among different detection methods. This enables users in various fields to select suitable algorithms and accelerate the research process.

**Figure 3:**
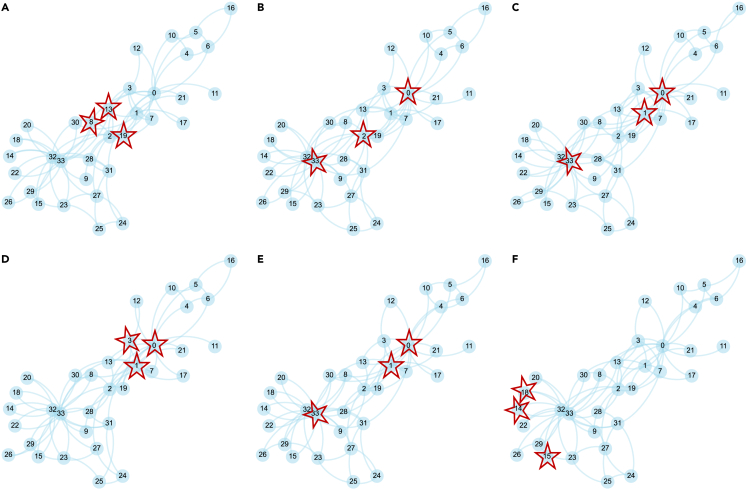
Visualization of SH spanner detection results for the Karate Club network Visualization of the results for the Karate Club network using different SH spanner detection algorithms: (A) HIS, (B) MaxD, (C) Greedy, (D) AP_Greedy, (E) NOBE_GA, and (F) BICC.

A visual representation of the results of different algorithms for detecting SH spanners is presented in Figure 3, where the nodes surrounded by pentagrams are the corresponding identified SH spanners for the Karate Club network. Users can easily capture an overview of the structure of a network and determine whether an SH spanner detection approach would work.

EasyGraph also implements representative network embedding algorithms, including DeepWalk,^58^ node2vec,^59^ LINE,^60^ and SDNE.^61^ These network embedding methods^58^^,^^59^^,^^60^^,^^61^ have been proven to be useful for maintaining inner network properties by vectorizing networks and their constituents, including nodes, edges, and subnetworks. It is noteworthy that these methods use different algorithms to calculate node similarity. They allow nodes that are similar in an original network to be close to each other in the low-dimensional representation space. With vectorized feature representations, various machine learning tasks associated with networks, including node classification, link prediction, community detection, and visualization, can be performed.

**Figure 4:**
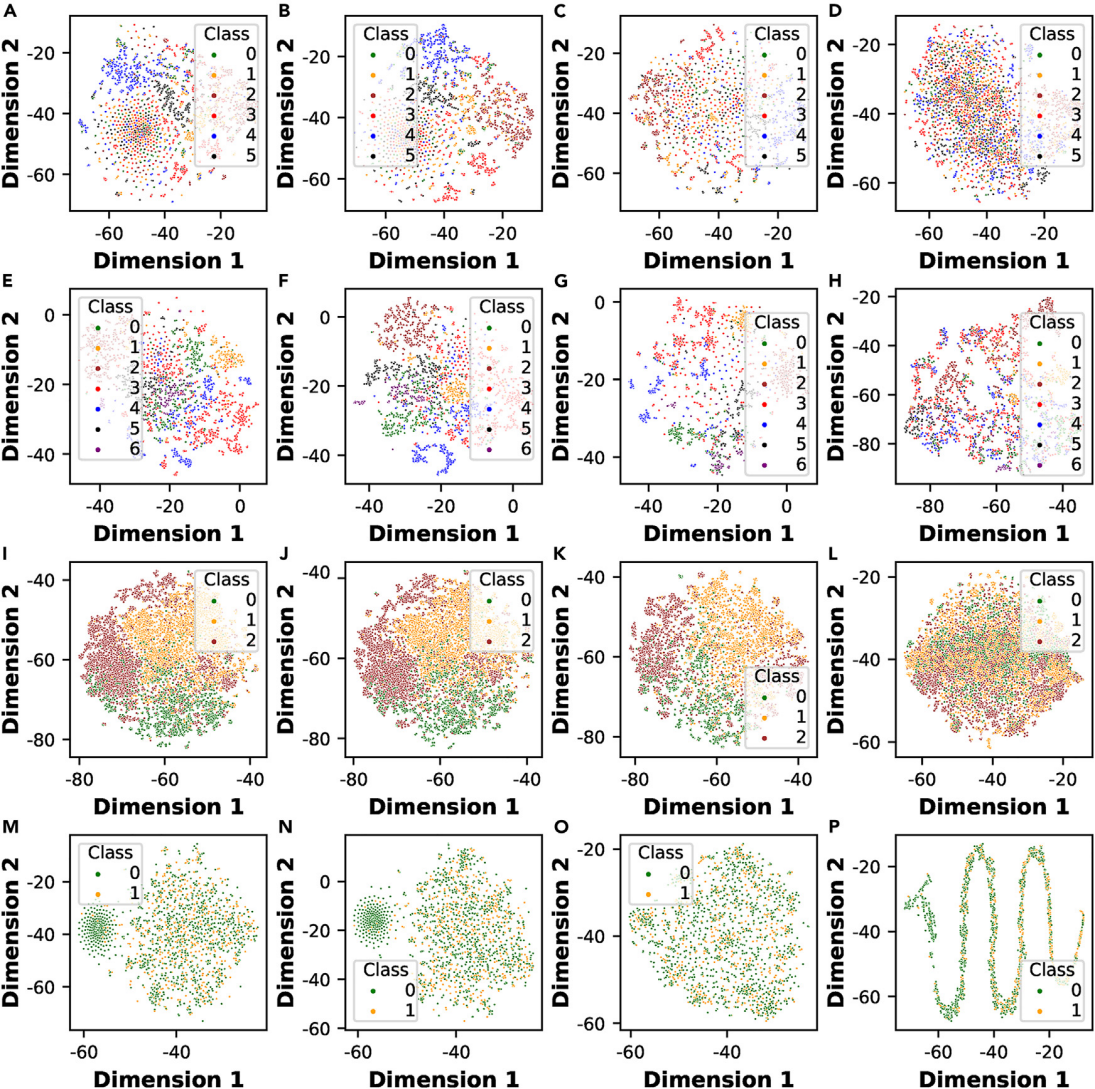
Visualization of the results of algorithms for network embedding using t-SNE for different network datasets (A–D) Visualization of the CiteSeer dataset using four network embedding algorithms: (A) DeepWalk, (B) node2vec, (C) LINE, and (D) SDNE. (E–H) Visualization of the Cora dataset using four network embedding algorithms: (E) DeepWalk, (F) node2vec, (G) LINE, and (H) SDNE. (I–L) Visualization of the PubMed dataset using four network embedding algorithms: (I) DeepWalk, (J) node2vec, (K) LINE, and (L) SDNE. (M–P) Visualization of the PPI dataset using four network embedding algorithms: (M) DeepWalk, (N) node2vec, (O) LINE, and (P) SDNE.

To demonstrate the various capabilities of embeddings generated by these four algorithms, we applied them to four representative network datasets, namely CiteSeer, Cora, PubMed, and PPI. The first three datasets are related to scientific publications, whereas the last dataset is a toy protein interaction network from the biology domain. Additional information regarding these datasets can be found at https://easy-graph.github.io/docs/reference/easygraph.datasets.html. Figure 4 presents visualization results based on different network embedding algorithms on four datasets using t-distributed stochastic neighbor embedding (t-SNE).^68^ The detailed parameter settings used for these algorithms are available in our source code. Notably, in these subfigures, one can clearly see that the embeddings of nodes with the same labels in the network are closer than those of nodes with different labels in two-dimensional space. LINE outperforms other methods on most datasets based on its unique ability to learn both first-order similarity and second-order similarity simultaneously. Additionally, the poor performance of SDNE might be attributed to the fact that it only takes an adjacency matrix as an input without considering node features and label information effectively.

### Performance enhancement of EasyGraph based on multiprocessing techniques and Python/C++ hybrid programming

Without losing generalization, we conducted comparisons on both random networks and real-world networks. All comparisons were conducted on a Linux server with an Inter Xeon CPU E5-2660 v.3 @ 2.60 GHz and 150 GB of RAM. Specifically, we utilized the classic Erdős-Rényi random network model^69^ to generate random networks with different scales ranging from 10,000 to 200,000 nodes. Additionally, twelve real-world network datasets from different disciplines were selected. The details of these datasets are presented in Table 2. We utilized igraph, which is a representative network analysis library, for comparison. In terms of time cost, the iteration times of all experiments ranged from 3 for directed networks to 5 for undirected networks. Please note that when executing the shortest path algorithm and closeness centrality function, we only utilized the same 1,000 nodes that were sampled for both igraph and EasyGraph on the real-world networks.

**Table 2:** Dataset information

Network	No. nodes	No. edges	Avg. degree	Density	is_directed
ER_10k_u	10,000	20,000	4.0	0.0004	false
ER_50k_u	50,000	100,000	4.0	$8.0\times 10^{-5}$	false
ER_100k_u	100,000	200,000	4.0	$4.0\times 10^{-5}$	false
ER_200k_u	200,000	400,000	4.0	$2.0\times 10^{-5}$	false
ER_10k_d	10,000	20,000	4.0	0.0002	true
ER_50k_d	50,000	100,000	4.0	$4.0\times 10^{-5}$	true
ER_100k_d	100,000	200,000	4.0	$2.0\times 10^{-5}$	true
ER_200k_d	200,000	400,000	4.0	$1.0\times 10^{-5}$	true
wiki-Vote	7,115	103,689	29.1466	0.0020	true
LastFM	7,624	27,806	7.2943	0.0010	false
ca-HepTh	9,877	25,998	5.2644	0.0005	false
p2p-Gnutella04	10,876	39,994	7.3545	0.0003	true
cd-HepPh	12,008	118,521	19.7403	0.0016	false
pgp	39,796	301,498	15.1521	0.0002	true
ca-CondMat	23,133	93,497	8.0834	0.0003	false
email-Enron	36,692	183,831	10.0202	0.0003	false
soc-Epinions1	75,879	508,837	13.4118	$8.8\times 10^{-5}$	true
soc-Slashdot0811	77,360	905,468	23.4092	0.0002	true
email-EuAll	265,214	420,045	3.1676	0.0003	true
web-NotreDame	325,729	1,497,134	9.1925	$1.5\times 10^{-7}$	true

Note that the word starting with “ER” in the first column of this table indicates that the network was generated by the Erdős-Rényi random network model. Additionally, the letter “u” at the end of the word indicates that the network is undirected, whereas the letter “d” indicates that the network is directed. The fourth column name, “Avg. degree,” refers to the value of the average degree of the network.

To investigate the advantages of multiprocessing, we selected different settings for the number of workers for comparison. Four representative functions implemented in EasyGraph, namely the local clustering coefficient,^70^ hierarchy,^62^ closeness centrality,^34^ and betweenness centrality^71^ functions, were examined. The local clustering coefficient is a metric that characterizes the local connections of a node for forming a cluster. For example, in the network of wiki-Vote, this metric refers to how closely Wikipedia users interact. The hierarchy metric can be used to measure the ability of nodes to serve as SH spanners. The closeness centrality and betweenness centrality are two useful metrics for identifying important nodes in a network.

**Figure 5:**
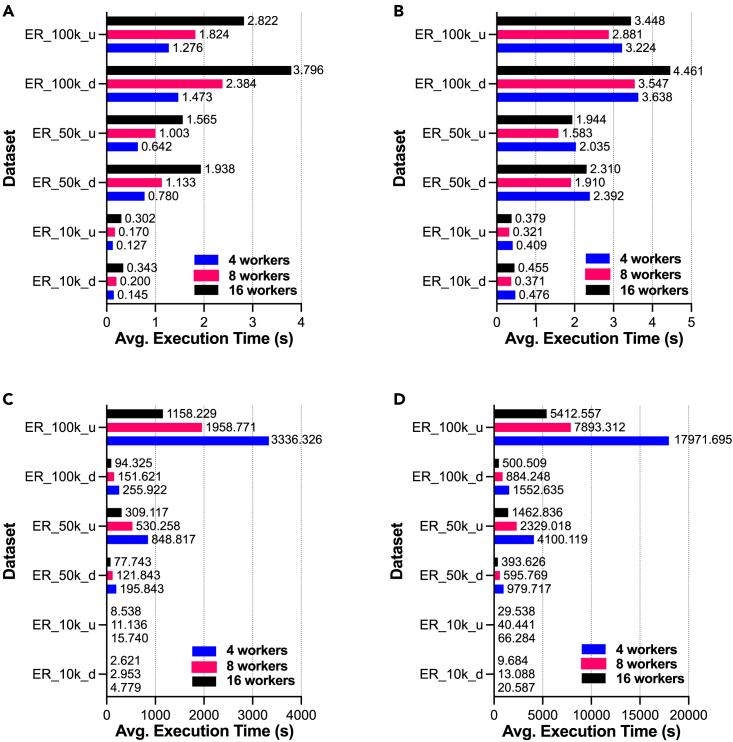
Advantages of multiprocessing in EasyGraph for functions on random networks Comparative analysis of EasyGraph with multiprocessing on random networks in terms of functions of (A) local clustering coefficient, (B) hierarchy, (C) closeness centrality, and (D) betweenness centrality.

**Figure 6:**
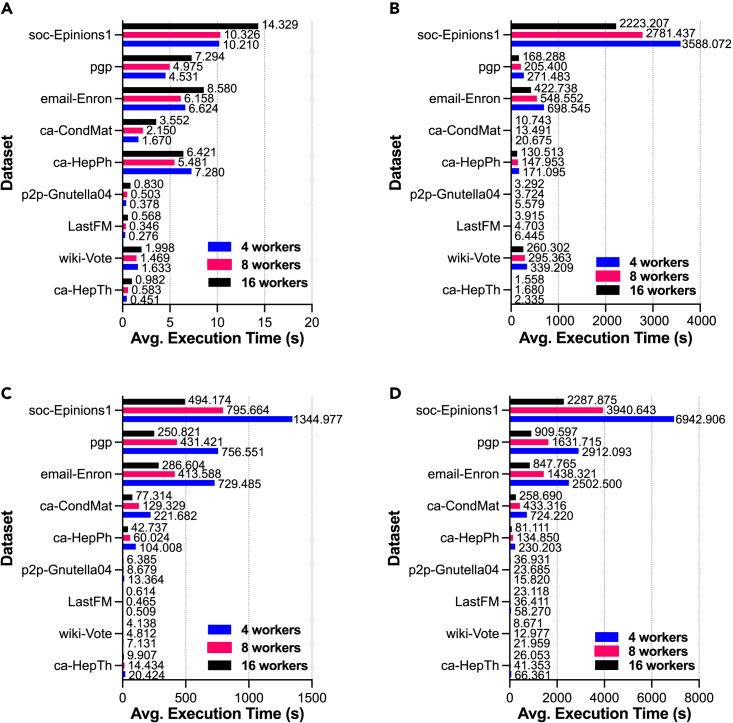
Advantages of multiprocessing in EasyGraph for functions on real-world networks Comparative analysis of EasyGraph with multiprocessing on real-world networks in terms of functions of (A) local clustering coefficient, (B) hierarchy, (C) closeness centrality, and (D) betweenness centrality.

Figures 5 and 6 present comparative analysis results for EasyGraph with multiprocessing on random networks and real-world networks, respectively. The vertical axes in the subfigures represent different networks, and the horizontal axes represent the time consumed by the corresponding functions. The bars with different colors in the figure indicate different settings for the number of workers. Interestingly, we found that not all network analysis algorithms would benefit from multiprocessing optimization techniques. Specifically, the multiprocessing technique significantly accelerated the calculation of metrics such as closeness centrality and betweenness centrality, whereas the calculation efficiency of the local clustering coefficient was not improved. The improvement of the hierarchy metric by the multiprocessing technique was inconsistent between random networks and real-world networks. One possible explanation is that multiprocessing is most useful when network analysis tasks can be split into small, independent subtasks that can be executed in parallel. Therefore, careful consideration of the trade-off between the benefits and costs of multiprocessing is necessary. For example, multiprocessing can be utilized to improve the efficiency of calculating closeness centrality, which is a key metric for network analysis. By implementing multiprocessing, such calculations can be distributed across multiple cores, thereby reducing computation time. However, the performance of multiprocessing for the local clustering coefficient metric is poor. One possible explanation could be the communication overhead between multiple processes. Additionally, the computational efficiency of multiprocessing techniques depends on the structure and characteristics of different networks.

To evaluate the advantages of Python/C++ hybrid programming, we considered igraph, which is a network library built on C/C++ code with Python interfaces, for comparisons of selected functions for both random networks and real-world networks. Specifically, we selected six classical functions, namely network loading, the multisource Dijkstra algorithm,^72^ betweenness centrality,^71^ closeness centrality,^34^ PageRank centrality,^41^ and k-core centrality.^73^ Network loading refers to the operation of loading network data and constructing network objects. The multisource Dijkstra algorithm is used to compute the shortest path through a set of source nodes in a network. Betweenness centrality,^71^ closeness centrality,^34^ PageRank centrality,^41^ and k-core centrality^73^ are four popular metrics that have been largely utilized to measure the importance of nodes in networks from different perspectives. For example, the metric of closeness centrality represents the extent to which a node is located at the center of a network. PageRank centrality is a metric used to rank a node by quantifying the importance of the nodes linked to it. K-core centrality is a metric that measures the relative importance of a node within a network based on k-core decomposition. Nodes with large core numbers are considered to be more important than those with lower values.

**Figure 7:**
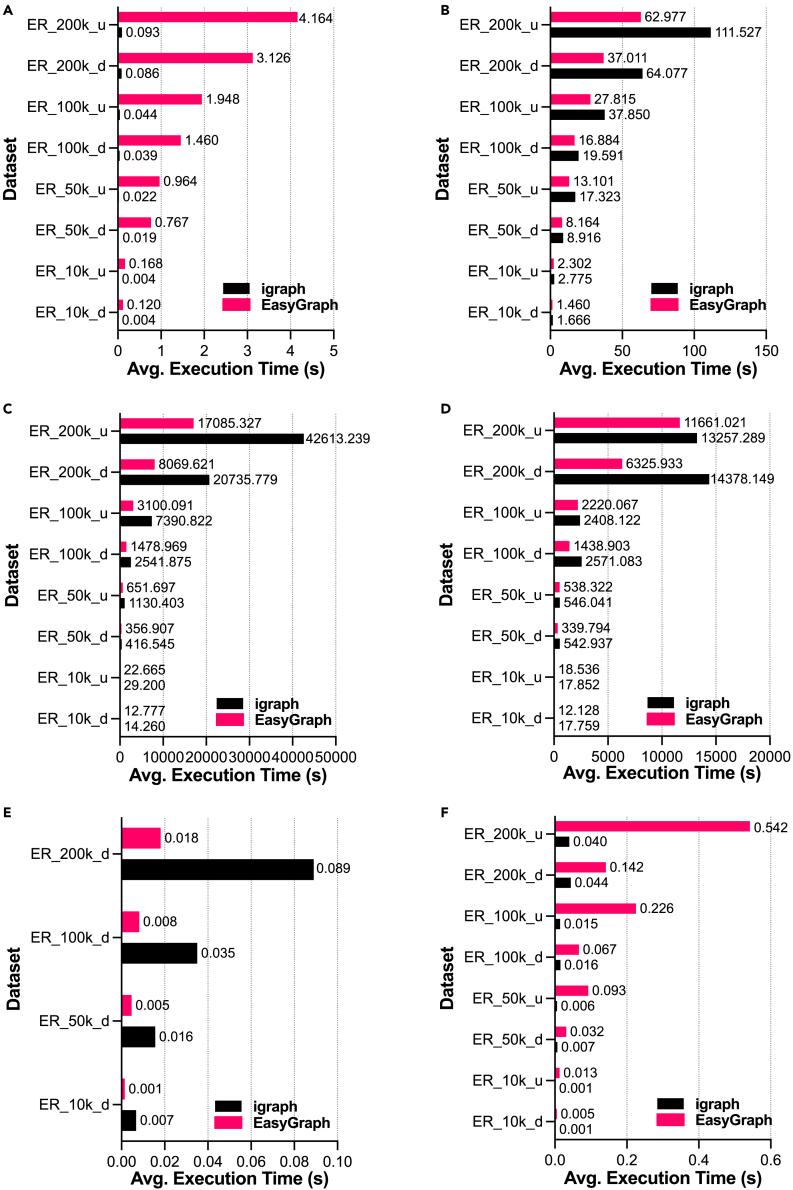
Advantages of hybrid programming in EasyGraph for functions on random networks Comparative analysis of EasyGraph with hybrid programming against igraph on random networks for various functions: (A) network loading, (B) multisource Dijkstra, (C) betweenness centrality, (D) closeness centrality, (E) PageRank centrality, and (F) k-core centrality.

Figure 7 presents a comparative analysis of EasyGraph with hybrid programming against igraph on random networks of varying scales. The vertical axes of all subfigures represent networks with different sizes, and the horizontal axes represent the time consumed by the corresponding function. Bars with different colors indicate the results of EasyGraph and igraph. Figure 7A reveals that the execution time of the network loading function in EasyGraph is longer than that of igraph, which can be attributed to EasyGraph’s consideration of the diversity of node types during the process of network construction. As a result, EasyGraph supports arbitrary hashable node types while still ensuring an acceptable time cost for network loading. In Figures 7B–7E, EasyGraph outperforms igraph because EasyGraph employs a series of optimization techniques, including the dirty flag design pattern,^74^ a singly linked list data structure,^75^ a segment tree data structure,^76^^,^^77^ and the Radix sorting algorithm.^78^ Specifically, the dirty flag design pattern^74^ generally refers to a programming technique that utilizes a flag to indicate the state of variables. In EasyGraph, we use a dirty flag to track the state of variables during network analysis. Therefore, EasyGraph writes updated values to memory only when the states of certain variables change, which could significantly reduce data writing operations and unnecessary overhead. The singly linked list^75^ is a data structure used to store information regarding adjacent nodes in a network. A singly linked list is advantageous when there is a need for efficient insertion and removal operations of nodes. For instance, network analysis algorithms that require the dynamic insertion or removal of nodes and edges can be enhanced by adopting a singly linked list data structure. A segment tree^76^^,^^77^ is a tree data structure that facilitates querying on segments of an array. In EasyGraph, we use a segment tree in Dijkstra’s algorithm to facilitate the identification of the shortest paths. Additionally, the Radix sorting algorithm^78^ is used to split integers into digits and then compare the integers by digits. In EasyGraph, we utilize this algorithm to optimize the k-core function.^73^ In Figure 7F, the performance of EasyGraph is inferior to that of igraph. This is because calculating the k-core centrality for random networks involves an additional step of transforming a singly linked list, which incurs additional running overhead.

**Figure 8:**
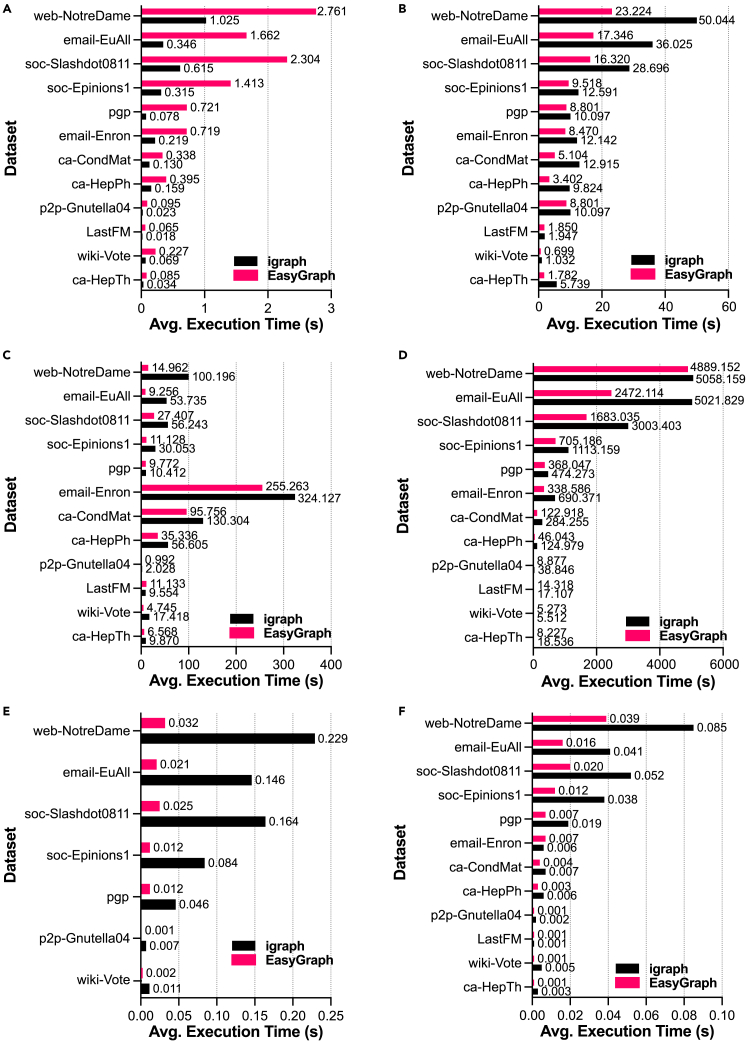
Advantages of hybrid programming in EasyGraph for functions on real-world networks Comparative analysis of EasyGraph with hybrid programming against igraph on real-world networks for various functions: (A) network loading, (B) multisource Dijkstra, (C) betweenness centrality, (D) closeness centrality, (E) PageRank centrality, and (F) k-core centrality.

Figure 8 presents a comparative analysis of EasyGraph with hybrid programming against igraph on real-world networks from different disciplines. In Figure 8A, one can see that the execution time of network loading in EasyGraph is slower than that in igraph. This is because EasyGraph adds a small amount of time overhead in exchange for good compatibility with different node types. In Figures 8B–8F, EasyGraph outperforms igraph because it is boosted by a series of optimization techniques, as described above. Furthermore, one can see that the functions implemented by EasyGraph still maintain an advantage on relatively large-scale networks such as email-EuAll and web-NotreDame. For example, in terms of analyzing closeness centrality on the email-EuAll network, EasyGraph saves almost 2,549 s over igraph, as indicated in Figure 8D. Regarding Figure 8F, the transformation of the singly linked list^75^ is conducted during the construction of the network data. Therefore, EasyGraph can directly calculate the k-core centrality for each node without additional transformation overhead.

Although EasyGraph is inferior to igraph in terms of the network loading function on all networks and k-core centrality on random networks, EasyGraph improves performance overall and achieves better compatibility on a series of important network analysis functions. Therefore, it is a powerful tool that users can use to perform network analysis tasks.

## Discussion

Network analysis is a highly interdisciplinary field that spans physics, chemistry, biology, mathematics, sociology, computer science, economics, and other disciplines. The universality of network data in different disciplines has made network analysis a valuable tool for exploring the commonalities and unique characteristics of various disciplines. With access to rich datasets from multiple domains, researchers have found some general properties, such as scale-free networks^8^^,^^79^ and small-world networks,^9^ that exist in various disciplines. Additionally, network analysis encompasses a number of significant research problems, including node centrality calculation, community detection, link prediction, SH spanner detection, network visualization, and information propagation in networks. These key research topics provide guidance and insights into problems in various disciplines. For example, the study of community detection has found applications in diverse domains, such as identifying fraud gangs in financial scenarios^80^^,^^81^ and public security fields.^82^ It has also been used to detect malicious accounts in online social networks.^83^^,^^84^ Furthermore, research on SH spanner detection has been applied to many types of networks, including social networks, supply chain networks, and road networks.^51^^,^^52^ Therefore, network analysis tools, such as NetworkX, igraph, and SNAP, have been developed and employed for research in different disciplines. These tools enable users to explore complex networks, identify important structures and features, and gain insights into the underlying dynamics of these networks.

Although there are numerous tools available for network analysis, some might have limitations that could potentially hinder their applicability for particular network data formats or network analysis tasks. Additionally, some tools might have inefficient implementations, which could potentially hinder their capability to utilize available computational resources effectively. Consequently, such limitations might increase execution time for large-scale networks. To address these problems, we proposed EasyGraph as an open-source toolkit designed to facilitate interdisciplinary network analysis. EasyGraph provides an easy-to-use interface and supports multiple formats of network data, which allows users to conduct network analysis rapidly without being limited by data formats. Furthermore, EasyGraph implements a range of significant functions for network analysis, including centrality measurement, community detection, SH spanner detection, motif detection, and network embedding. Additionally, EasyGraph has been implemented with hybrid programming (using Python and C++) and leverages multiprocessing techniques to enhance the efficiency of network analysis further. We demonstrated the performance benefits of EasyGraph on large-scale networks from different disciplines. By overcoming the limitations of existing network analysis tools, EasyGraph provides a powerful and flexible solution for users across a range of fields to conduct effective interdisciplinary network analysis.

Several improvements can be made to our work. First, to enhance compatibility with different types of networks, we plan to extend EasyGraph to support bipartite networks,^85^^,^^86^ heterogeneous networks,^87^^,^^88^ dynamic networks,^89^ and higher-order networks.^90^^,^^91^ We believe that supporting these important but less frequently used types of networks will help users construct networks in a more flexible manner. Second, we will continue to track and implement methods from the latest studies on network analysis functions so that EasyGraph can continuously serve as a benchmark platform for users to compare different network analysis functions. This will enable us to keep pace with new developments in the field and provide users with state-of-the-art tools for network analysis. Finally, we will strive to deploy EasyGraph as a cloud-based web service to help users who are not familiar with Python programming perform network analysis easily. By making EasyGraph more accessible to a wider audience, we hope to encourage more users to leverage the power of EasyGraph in their work.

### Limitations of the study

In this section, we discuss the limitations of EasyGraph. Our goal is to provide users with a comprehensive view of the available options for network analysis and highlight the unique features of EasyGraph.

First, EasyGraph does not currently support some less-popular functions such as *is_isomorphic*, *LFR_benchmark_graph*, and *hypercube_graph*, which NetworkX has already implemented. Therefore, NetworkX might be a good fit if a user currently wishes to use these functions. Additionally, we are continuously expanding EasyGraph to provide more algorithm choices. Users and developers are encouraged to participate in the development of EasyGraph.

Second, EasyGraph does not currently focus on functions for comprehensive visualization, which are provided by Gephi and Cytoscape. Network visualization allows users to understand the intrinsic relationships and structures of network data. Therefore, we plan to develop an interactive visualization interface that facilitates data exploration and analysis to derive valuable insights from network data. Gephi and Cytoscape might still be good choices if a user currently requires network visualization.

Third, in its current design, EasyGraph supports basic types of networks such as directed and undirected networks and weighted and unweighted networks, as well as multigraphs. However, it has not yet incorporated some advanced network types such as dynamic networks, bipartite networks, heterogeneous networks, and higher-order structures. For these types of networks, specialized packages such as HyperNetX (https://github.com/pnnl/HyperNetX) for hypergraphs and GraphStream (https://graphstream-project.org/doc/) for dynamic networks are more suitable.

Therefore, users are advised to choose appropriate network analysis tools according to their requirements and the scale of their network datasets. The functions and usage scenarios supported by EasyGraph as well as other network analysis libraries have been summarized in Table 3. NetworkX is a promising option for processing small-scale network data and offers a range of convenient features such as simple plots and algorithm implementations. The igraph library provides some efficient algorithms and visualization tools for large-scale network data. The graph-tool library focuses on the statistical analysis of networks. Gephi and Cytoscape both excel at visualization of network data. Notably, EasyGraph supports more formats of network data than other tools. It offers important specialized functions such as SH spanner detection and graph embedding. For networks that contain more than thousands of nodes, EasyGraph outperforms other tools on several important network analysis tasks, including algorithms for computing the shortest paths, PageRank centrality, betweenness centrality, closeness centrality, and k-core centrality.

**Table 3:** Feature comparisons between EasyGraph and other network analysis libraries

Features	Tools	EasyGraph	NetworkX	igraph	SNAP	graph-tool
Network I/O	Edge List	✓	✓	✓	✓	–
	GML	✓	✓	✓	–	✓
	GraphML	✓	✓	✓	–	✓
	Pajek	✓	✓	✓	–	–
	GraphViz	✓	–	✓	✓	✓
Centrality calculation	degree	✓	✓	✓	✓	✓
	betweenness centrality	✓	✓	✓	✓	✓
	closeness centrality	✓	✓	✓	✓	✓
	Katz centrality	–	✓	✓	✓	✓
Structural hole spanner detection	effective size	✓	✓	–	–	–
	hierarchy	✓	–	–	–	–
	HIS	✓	–	–	–	–
	AP_Greedy	✓	–	–	–	–
Community detection	Girvan-Newman	✓	✓	✓	✓	✓
	Louvain	–	✓	✓	✓	✓
	label propagation	✓	✓	✓	✓	✓
	InfoMAP	–	✓	✓	✓	✓
Network visualization	matplotlib	✓	✓	✓	✓	✓
	plotly	–	–	✓	–	–
	Cairo	–	–	✓	–	✓
	GTK+	–	–	✓	–	✓
Network embedding	DeepWalk	✓	–	–	–	–
	node2vec	✓	–	–	✓	–
	LINE	✓	–	–	–	–
	SDNE	✓	–	–	–	–

## Experimental procedures

### Resource availability

#### Lead contact

Additional information and requests for resources should be directed to and will be fulfilled by the lead contact, Yang Chen (chenyang@fudan.edu.cn).

#### Materials availability

This study did not generate any new materials.

## Data Availability

Source code and package installation instructions can be found at Zenodo (https://doi.org/10.5281/zenodo.8041952).^92^ Datasets for network embedding can be constructed from https://zenodo.org/record/8041952,^92^ and datasets for benchmarking are available at Zenodo (https://zenodo.org/record/8042042^93^).
